# Crohn’s disease and short bowel syndrome

**DOI:** 10.1007/s00595-022-02545-0

**Published:** 2022-06-29

**Authors:** Philippe Pinton

**Affiliations:** Translational Medicine and Clinical Pharmacology, Ferring Pharmaceuticals A/S, International PharmaScience Center, Amager Strandvej 405, 2770 Kastrup, Denmark

To the Editor,

Mizushima et al. recently published remarkable explorative data on the causes, treatment patterns and outcomes of short bowel syndrome with intestinal failure (SBS-IF) in Japanese adult patients [1]. The observed outcomes demonstrate the critical remaining medical and therapeutic needs for these patients.

Overall, the characteristics of these Japanese adults with SBS-IF are very similar to what is observed elsewhere in the world but they notably differ from those of the small population included in a contemporary clinical trial (CT) evaluating the efficacy and safety of a glucagon-like-peptide-2 analog [2], in that the mean age in the CT was 40.9 ± 12.4 years versus 61.4 ± 17.3 years in Mizushima’s real-world (RW) study. Weaning from parenteral nutrition (PN) declines with age. Crohn’s disease (CD) was the cause of SBS-IF in 72.7% of the patients in the CT in comparison to 20.1% of the patients in the RW study. In Japan, biologics are recommended to treat moderate-to-severe CD. A mirror image study using an insurance database showed that 51% of patients with CD are on biologics [3].

Observed differences in patients’ characteristics support the discussion raised by Mizushima et al. (1) CD is typically diagnosed in early adulthood; (2) CD surgeries are usually performed in late age and after a long disease duration; and (3) biologics reduce the rate of surgery, preserve the residual bowel and facilitate earlier weaning from PN.

SBS is the most common cause of chronic intestinal failure (IF) in CD [4]. Risk factors for and characteristics of IF in Japanese patients with CD have been previously identified [4–6]. These include cumulative initial inflammation, short residual small intestinal length, and non-use of anti-tumor necrosis factor-alpha therapy.

Detailed data about the features of CD and administered treatments (e.g., fluids, biologics, or teduglutide) would help to provide a better picture of SBS-IF in Japan.

These differences between RW and CT data in Japanese adult patients with SBS also perfectly illustrate how real-world data and randomized control trial data can be mutually complementary and how the first can improve the conclusions of clinical trial by better tackling the variety and heterogeneity of populations.
